# Epidemiology and prognosis of anti-infective therapy in the ICU setting during acute pancreatitis: a cohort study

**DOI:** 10.1186/s13054-019-2681-5

**Published:** 2019-12-05

**Authors:** Philippe Montravers, Elie Kantor, Jean-Michel Constantin, Jean-Yves Lefrant, Thomas Lescot, Nicolas Nesseler, Catherine Paugam, Matthieu Jabaudon, Hervé Dupont

**Affiliations:** 1 Département d’Anesthésie-Réanimation, CHU Bichat-Claude Bernard, HUPNVS, APHP, 48 rue Henri Huchard, F-75018 Paris, France; 2 Université de Paris, Paris, France; 3 INSERM UMR 1152 – Université de Paris, Paris, France; 4 Département de Médecine Post-opératoire, CHU Clermont-Ferrand, Clermont-Ferrand, France; 5 Université Clermont Auvergne, CNRS UMR 6293, INSERM U1103, GReD, Clermont-Ferrand, France; 6 Division of Anaesthesiology, Critical Care, Pain and Emergency Medicine, Nîmes University Hospital, and EA 2992, Université Montpellier, Nîmes, France; 7 Department of Anaesthesia and Critical Care, Saint-Antoine University Hospital, Assistance Publique-Hôpitaux de Paris, and Sorbonne Universités, UPMC Univ Paris 06, Paris, France; 8 Surgical Intensive Care Unit, Hôpital Pontchaillou, and Inserm U 991, Université de Rennes 1, Rennes, France; 9 Department of Anaesthesiology and Critical Care Medicine, Hôpital Beaujon, Assistance Publique-Hôpitaux de Paris, Paris, France; 10 Medical and Surgical ICU, Amiens University Hospital and INSERM U1088, University of Picardy Jules Verne, Amiens, France

**Keywords:** Acute pancreatitis, Intensive care unit, Anti-infective therapy, Carbapenems, Mortality

## Abstract

**Background:**

Recent international guidelines for acute pancreatitis (AP) recommend limiting anti-infective therapy (AIT) to cases of suspected necrotizing AP or nosocomial extrapancreatic infection. Limited data are available concerning empirical and documented AIT prescribing practices in patients admitted to the intensive care unit (ICU) for the management of AP.

**Methods:**

Using a multicentre, retrospective (2009–2014), observational database of ICU patients admitted for AP, our primary objective was to assess the incidence of AIT prescribing practices during the first 30 days following admission. Secondary objectives were to assess the independent impact of centre characteristics on the incidence of AIT and to identify factors associated with crude hospital mortality in a logistic regression model.

**Results:**

In this cohort of 860 patients, 359 (42%) received AIT on admission. Before day 30, 340/359 (95%) AIT patients and 226/501 (45%) AIT-free patients on admission received additional AIT, mainly for intra-abdominal and lung infections. A large heterogeneity was observed between centres in terms of the incidence of infections, therapeutic management including AIT and prognosis. Administration of AIT on admission or until day 30 was not associated with an increased mortality rate. Patients receiving AIT on admission had increased rates of complications (septic shock, intra-abdominal and pulmonary infections), therapeutic (surgical, percutaneous, endoscopic) interventions and increased length of ICU stay compared to AIT-free patients. Patients receiving delayed AIT after admission and until day 30 had increased rates of complications (respiratory distress syndrome, intra-abdominal and pulmonary infections), therapeutic interventions and increased length of ICU stay compared to those receiving AIT on admission. Risk factors for hospital mortality assessed on admission were age (adjusted odds ratio [95% confidence interval] 1.03 [1.02–1.05]; *p* < 0.0001), Balthazar score E (2.26 [1.43–3.56]; *p* < 0.0001), oliguria/anuria (2.18 [1.82–4.33]; *p* < 0.0001), vasoactive support (2.83 [1.73–4.62]; *p* < 0.0001) and mechanical ventilation (1.90 [1.15–3.14]; *p* = 0.011), but not AIT (0.63 [0.40–1.01]; *p* = 0.057).

**Conclusions:**

High proportions of ICU patients admitted for AP receive AIT, both on admission and during their ICU stay. A large heterogeneity was observed between centres in terms of incidence of infections, AIT prescribing practices, therapeutic management and outcome. AIT reflects the initial severity and complications of AP, but is not a risk factor for death.

## Introduction

Limited data are available concerning empirical and documented anti-infective therapy (AIT) prescribing practices in patients admitted to the intensive care unit (ICU) for management of acute pancreatitis (AP). Many clinical conditions related to abdominal or extra-abdominal sources of infection can lead to the prescription of AIT. Recent international guidelines recommend limiting the use of antibiotics (AB) to cases of suspected necrotizing pancreatitis or nosocomial extra-pancreatic infection and to treat other known fungal infections with antifungal therapy (AF) [1, 2].

Most publications focusing on AIT in patients with AP have reported single-centre experiences [3–8], while multicentre data on the clinical and microbiological features of acute infections in ICU patients are rare. The largest multicentre point-prevalence study collected data from ICU patients one decade ago during the EPIC II trial [9]. The authors reported that half of these patients were infected and 71% received antibiotics on the day of the study.

We used data from a large multicentre retrospective database of ICU patients with AP [10] to describe AIT use on ICU admission and during the first 30 days, evaluate between-centre variability in terms of the incidence of infections and AIT prescribing practices, evaluate outcome in terms of morbidity and mortality and identify independent risk factors evaluated on admission associated with mortality.

## Methods

Patient data were extracted from a multicentre, retrospective, observational database involving 17 French and Belgian ICUs [10]. Patients for whom data concerning AIT with curative intent were available from ICU admission until day 30 were selected. AIT administered before ICU admission could not be determined.

This study was approved by the French Society of Anaesthesiology and Critical Care Medicine Ethics Committee (00010254-2015-017) and the French Personal Data Protection Agency (16–023). According to French legislation, this observational study did not require the patients’ informed consent. The study was performed in accordance with the STROBE recommendations [10].

### Clinical data

Baseline demographic, clinical and laboratory characteristics; organ failure; AIT; and organ support therapies were recorded on ICU admission and until day 30. Diagnostic criteria for sepsis and septic shock were those used at the time of admission of the patients in agreement with the International Sepsis Definitions Conference [11]. Organ failures, scored according to the SOFA score, were used to describe severity on ICU admission [12]. Commonly reported risk factors for AP [1] and Balthazar score were assessed on admission, while the BISAP score was calculated retrospectively [13]. Clinical management, microbiological examinations, criteria for diagnosis of infection and selection of AIT were decided according to local protocols based on the recommendations of the French Society of Anaesthesiology and Critical Care Medicine [14].

We analysed patients receiving AIT on ICU admission (day 0) and during the first 30 days (day > 0 to day 30) of management, and AIT duration was recorded. Patients who did not receive any AIT at any time during the 30-day follow-up period were classified as AIT-free. AIT with curative intent on day 0 was defined as empirical or documented AIT [15], and the type of antibiotic (beta-lactams, carbapenems, aminoglycosides, and anti-Gram-positive agents) and antifungal (echinocandins and azoles) therapies were analysed on day 0 and during the first 30 days.

### Outcomes

The primary objective of our study was to assess the incidence of AIT during the first 30 days following admission for AP. Secondary objectives were to assess the following: (i) the independent impact of centre characteristics on the incidence of AIT, (ii) all-cause mortality at hospital discharge and (iii) factors associated with crude hospital mortality.

In line with these objectives, the primary study endpoints were the proportions of patients receiving AIT during the first 30 days following admission, with a focus on pancreatic and extra-pancreatic infections and AIT use across specific conditions. Secondary endpoints were infectious complications of AP (e.g. organ failure, sepsis, surgical complications), therapeutic interventions (surgical, percutaneous, endoscopic) from baseline to day 30, all-cause mortality at hospital discharge and assessment of risk factors of death.

### Statistical analysis

Results are expressed as median and interquartile range [IQR] or number and proportions. The chi-square test and Fisher’s exact test were used to compare discrete variables, and unpaired Wilcoxon tests were used to compare quantitative variables.

The purpose of this study was strictly exploratory. We therefore chose not to take inflation of the alpha risk into account. For the same purpose, only one multivariate model was constructed for the overall population to investigate the association between mortality and the variables of interest. Risk factors for death were assessed by univariate analysis, and unadjusted odds ratio (OR) and 95% confidence intervals (CI) were calculated. Variables with *p* < 0.10 on univariate analysis were introduced as predictive factors into the complete-case multivariate logistic regression analyses using a backward selection method. The centre effect and AIT on ICU admission were forced into these analyses. The collinearity between predictors was analysed, but no sensitivity analyses were performed. The BISAP score was not used in these analyses because of its post hoc calculation. A logistic model was evaluated for discrimination with the C-statistic and for calibration with the Hosmer-Lemeshow test. No special treatment was performed for missing variables. Statistical analysis was performed with SAS© 9.4 (SAS Institute, Cary, NC, USA).

## Results

### Study population

**Fig. 1:**
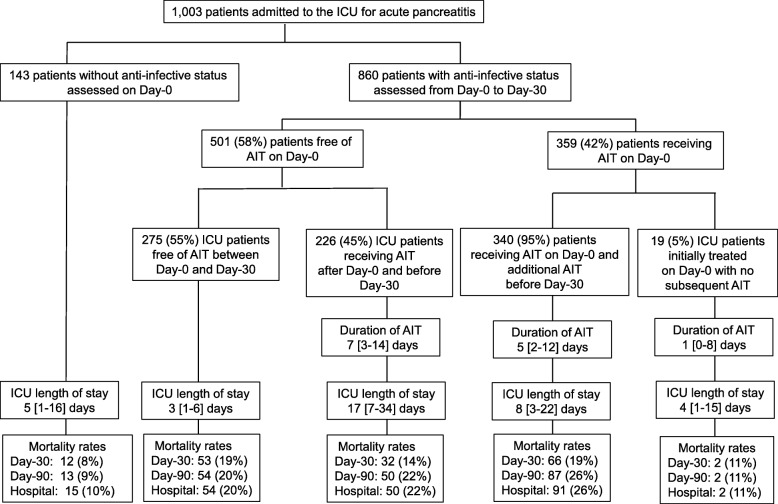
Flow chart of the study population

Over a 50-month period (2009–2014), individual data for 1003 patients with a diagnosis of AP were collected from medical records on ICU admission and for the first 30 days of ICU stay or until early discharge or death. From this cohort, 860 patients for whom information on AIT was available were analysed (Fig. 1). Overall, 275 (32%) patients did not receive any AIT during their ICU stay. These patients had a short ICU stay and predominantly presented less severe disease than patients receiving AIT (Table 1).

**Table 1:** Clinical features and prognosis for the AIT-free patients and those receiving AIT

	Missing data	AIT-free patients *n* = 275	Patients receiving AIT during their ICU stay *n* = 585	*p* value
Male, *n* (%)	1/4	166 (61)	386 (66)	NS
Age, years, median [IQR]	3/4	55 [40–71]	59 [48–72]	< 0.01
Underlying diseases				
Diabetes, *n* (%)	0	45 (16)	91 (16)	NS
Cardiovascular disease, *n* (%)	0	157 (57)	342 (58)	NS
Respiratory disease, *n* (%)	0	44 (16)	100 (17)	NS
Renal disease, *n* (%)	0	24 (9)	31 (5)	NS
Liver disease, *n* (%)	0/1	30 (11)	63 (11)	NS
Smoking, *n* (%)	0	137 (50)	251 (43)	NS
Alcoholism, *n* (%)	0/1	128 (47)	247 (42)	NS
Attributable cause of pancreatitis				
Alcoholism, *n* (%)	0	80 (29)	146 (25)	NS
Gallstones, *n* (%)	0	97 (35)	247 (42)	NS
Post-ERCP, *n* (%)	0	9 (3)	47 (8)	< 0.01
Hypertriglyceridaemia, *n* (%)	0	26 (9)	31 (5)	< 0.05
Cancer, *n* (%)	0	8 (3)	6 (1)	NS
Toxic, *n* (%)	0	4 (1)	17 (3)	NS
Undetermined, *n* (%)	0	51 (19)	96 (16)	NS
Clinical presentation on admission				
Pain onset/ICU admission interval, days, median [IQR]	0	2 [1–3]	3 [1–8]	< 0.001
Oliguria/anuria, *n* (%)	0	120 (44)	253 (43)	NS
BISAP score, median [IQR]	12/22	1 [0–1]	1 [1–2]	< 0.001
Balthazar score E, *n* (%)	0	137 (50)	356 (61)	< 0.01
Severity criteria on admission				
SOFA score, median [IQR]	9/14	3 [1–5]	5 [3–7]	< 0.001
Respiratory failure*, *n* (%)	0	39 (14)	207 (35)	< 0.001
Cardiovascular failure*, *n* (%)	0	37 (13)	202 (35)	< 0.001
Renal failure*, *n* (%)	0	43 (16)	100 (17)	NS
Septic shock, *n* (%)	0	3 (1)	114 (19)	< 0.001
Acute respiratory distress syndrome, *n* (%)	0	9 (3)	53 (9)	< 0.01
Therapeutic management on admission				
Vasoactive support, *n* (%)	0/1	38 (14)	207 (35)	< 0.001
Mechanical ventilation, *n* (%)	1/5	38 (14)	187 (32)	< 0.001
Fluid loading, *n* (%)	2/12	178 (65)	447 (78)	< 0.001
Renal replacement therapy, *n* (%)	1/10	16 (6)	62 (11)	< 0.05
Main treatments between day > 0 and day 30				
Need for red blood cell transfusion, *n* (%)	0	31 (11)	195 (33)	< 0.001
Number of days of mechanical ventilation	1/2	0 [0–0]	4 [0–16]	< 0.001
Vasoactive support, *n* (%)	0/1	59 (21)	335 (57)	< 0.001
Renal replacement therapy, *n* (%)	1/1	42 (15)	178 (30)	< 0.001
Duration of RRT, days, median [IQR]	14/0	2 [2–4]	7 [2–15]	< 0.001
Main complications between day > 0 and day 30				
Acute respiratory distress syndrome, *n* (%)	0/2	40 (15)	201 (34)	< 0.001
Septic shock, *n* (%)	24/47	4 (2)	210 (39)	< 0.001
Pancreatic necrosis, *n* (%)	12/18	164 (62)	393 (69)	< 0.05
Infected necrosis, *n* (%)	15/29	5 (2)	200 (36)	< 0.001
Gastro-intestinal perforation, *n* (%)	12/32	4 (1)	50 (9)	< 0.001
Vascular thrombosis, *n* (%)	12/26	22 (8)	86 (15)	< 0.01
Acute mesenteric ischaemia, *n* (%)	12/28	25 (10)	63 (11)	NS
Intra-abdominal collection, *n* (%)	10/22	37 (14)	242 (43)	< 0.001
Abdominal compartment syndrome, *n* (%)	12/26	17 (6)	66 (12)	< 0.05
Haemorrhage, *n* (%)	11/26	19 (7)	81 (14)	< 0.01
Peritonitis, *n* (%)	12/30	3 (1)	85 (15)	< 0.001
Cholangitis, *n* (%)	15/25	2 (1)	57 (10)	< 0.001
Digestive fistula, *n* (%)	12/28	1 (1)	30 (5)	< 0.001
Clinical management between day > 0 and day 30				
Endoscopic necrosectomy, *n* (%)	2/6	24 (9)	152 (26)	< 0.001
Surgical necrosectomy, *n* (%)	0/6	32 (12)	219 (38)	< 0.001
Radiological drainage, *n* (%)	0/6	8 (3)	120 (21)	< 0.001
Duration of ICU stay, days, median [IQR]	0	3 [1–6]	12 [4–27]	< 0.001
ICU readmission, *n* (%)	0/1	6 (2)	50 (9)	< 0.001
Hospital mortality rate, *n* (%)	0	54 (20)	143 (24)	NS
Time to death, days, median [IQR]	0	1.5 [1–2]	12 [2–34]	< 0.001

*NS* non-significant, *NA* not applicable, *ERCP* endoscopic retrograde cholangiopancreatography

*According to the definition of the SOFA score

### Anti-infective therapy on ICU admission

At the time of ICU admission, 359/860 (42%) patients were receiving AIT with curative intent, while no cases of prophylactic AIT were reported (Fig. 1). No difference was observed between patients receiving AIT on day 0 and AIT-free patients on admission in terms of either demographic data or risk factors for AP, while patients receiving AIT presented criteria of more severe disease and more intensive baseline therapy (Table 2).

**Table 2:** Clinical features for the patients with/without anti-infective therapy on ICU admission (day 0)

	Missing data	AIT-free patients on day 0 *n* = 501	Patients receiving AIT on day 0 *n* = 359	*p* value
Male, *n* (%)	0/5	313 (62)	239 (68)	NS
Age, years, median [IQR]	3/4	56 [43–71]	60 [49–73]	< 0.01
Clinical presentation on admission				
Pain onset/ICU admission interval, days, median [IQR]	0	2 [1–4]	4 [1–11]	< 0.001
Oliguria/anuria, *n* (%)	0	219 (44)	154 (43)	NS
BISAP score, median [IQR]	16/18	1 [0–1]	2 [1–2]	< 0.001
Balthazar score E, *n* (%)	0	293 (58)	200 (56)	NS
Severity criteria on admission				
SOFA score, median [IQR]	10/12	4 [2–6]	5 [3–7]	< 0.001
Respiratory failure*, *n* (%)	0	109 (22)	137 (38)	< 0.001
Cardiovascular failure*, *n* (%)	0	77 (15)	162 (45)	< 0.001
Renal failure*, *n* (%)	0	90 (18)	53 (15)	NS
Septic shock, *n* (%)	0	7 (1)	110 (31)	< 0.001
Acute respiratory distress syndrome, *n* (%)	0	25 (5)	37 (10)	< 0.01
Therapeutic management on admission				
Vasoactive support, *n* (%)	0/1	80 (16)	165 (46)	< 0.001
Mechanical ventilation, *n* (%)	1/5	95 (19)	130 (37)	< 0.001
Fluid loading, *n* (%)	2/12	348 (70)	277 (80)	< 0.01
Renal replacement therapy, *n* (%)	1/10	36 (7)	42 (12)	< 0.05
Anti-infective therapy, *n* (%)	0	–	359 (100)	< 0.001
Main treatments between day > 0 and day 30				
Need for red blood cell transfusion, *n* (%)	0	110 (22)	116 (32)	< 0.001
Number of days of mechanical ventilation	2/8	0 [0–9]	2 [0–10]	< 0.001
Vasoactive support, *n* (%)	0/1	186 (37)	208 (58)	< 0.001
Renal replacement therapy, *n* (%)	2/0	127 (25)	93 (26)	NS
Duration of RRT, days, median [IQR]	6/9	5 [2–14]	5 [2–15]	NS
Main complications between day > 0 and day 30				
Acute respiratory distress syndrome, *n* (%)	0/2	142 (28)	99 (28)	NS
Septic shock, *n* (%)	39/32	90 (19)	124 (38)	0.001
Pancreatic necrosis, *n* (%)	13/17	339 (69)	218 (64)	NS
Infected necrosis, *n* (%)	20/24	88 (18)	117 (35)	< 0.001
Gastro-intestinal perforation, *n* (%)	20/24	27 (6)	27 (8)	NS
Vascular thrombosis, *n* (%)	16/22	55 (11)	53 (16)	NS
Acute mesenteric ischaemia, *n* (%)	18/22	52 (11)	36 (11)	NS
Intra-abdominal collection, *n* (%)	14/18	127 (26)	152 (45)	< 0.001
Abdominal compartment syndrome, *n* (%)	16/22	52 (11)	31 (9)	NS
Haemorrhage, *n* (%)	16/21	53 (11)	47 (14)	NS
Peritonitis, *n* (%)	19/23	29 (6)	59 (18)	< 0.001
Cholangitis, *n* (%)	21/19	18 (4)	41 (12)	< 0.001
Digestive fistula, *n* (%)	19/21	11 (2)	20 (6)	< 0.01
Clinical management between day > 0 and day 30				
Endoscopic necrosectomy, *n* (%)	3/5	89 (18)	87 (25)	< 0.05
Surgical necrosectomy, *n* (%)	1/5	119 (24)	132 (37)	< 0.001
Radiological drainage, *n* (%)	1/5	56 (11)	72 (20)	< 0.001
Duration of ICU stay, days, median [IQR]	0	6 [2–17]	8 [3–21]	< 0.05
ICU readmission *n* (%)	0/1	30 (6)	26 (7)	NS
Hospital mortality rate, *n* (%)	0	104 (21)	93 (26)	NS
Time to death, days, median [IQR]	0	3 [1–18]	7 [1–31]	NS

*NS* non-significant, *NA* not applicable, *AIT* anti-infective therapy, *ERCP* endoscopic retrograde cholangiopancreatography

*According to the definition of the SOFA score

Most patients (*n* = 299/359 (83%)) with AIT on day 0 received empirical AB therapy, while 60 (17%) cases received documented AB therapy (Additional file 3: Table S1), with marked between-centre variability (range 9–64%). In three centres, more than 25% of cases were receiving documented AIT on day 0. Patients receiving documented AB had less severe disease than those receiving empirical AIT, but they received similar proportions of broad-spectrum AB and higher proportions of AF (Additional file 3: Table S1).

**Fig. 2:**
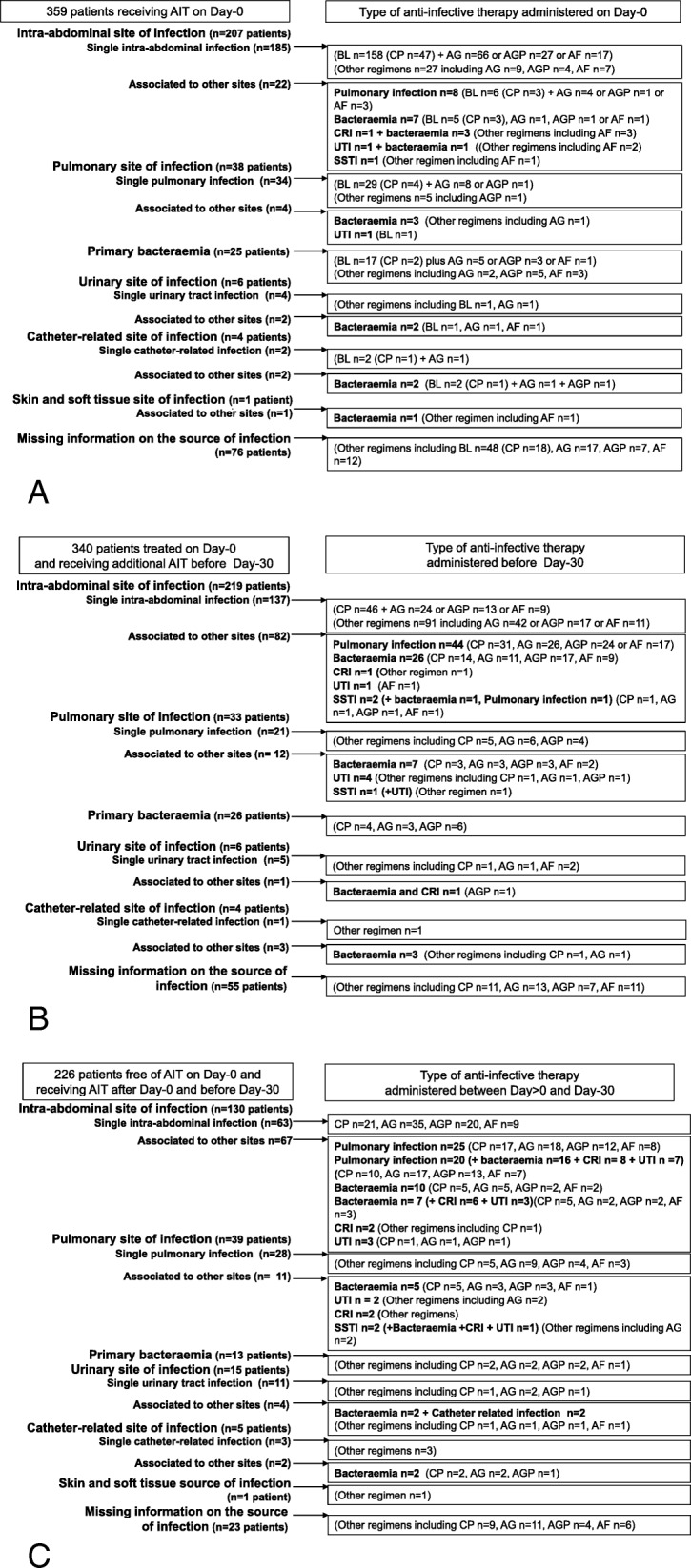
Most frequent sources of infection recorded and treated on admission in 359 patients (**a**), during the 30 days of follow-up for 340 of them (**b**) and in another 226 AIT-free patients on admission treated after day 0 and before day 30 (**c**). AF, antifungal therapy; AG, aminoglycosides; AGP, anti-Gram-positive agents; BL, beta-lactams; CP, carbapenems; CRI, catheter-related infection; UTI, urinary tract infection; SSTI, skin and soft tissue infection

On day 0, the indications for AIT varied among centres (Additional file 1: Figure S1). The predominant indications for AIT were intra-abdominal infections (*n* = 207 patients, including 173 (58%) receiving empirical therapy), pulmonary infections (*n* = 46, empirical therapy *n* = 41 (14%)) and bacteraemia (*n* = 43, empirical therapy *n* = 28 (9%)). Among the other sources of infections, low proportions of catheter-related infections, urinary tract infections and skin and soft tissue infections were also recorded (Fig. 2a). Overall, mixed sources of infection in patients with intra-abdominal infections were reported in 22 (11%) cases, including 11 bacteraemic intra-abdominal infections and 8 cases with combined intra-abdominal and pulmonary infections (Fig. 2a). Among the 46 (13%) cases initially treated for pneumonia, 3 cases of bacteraemia were observed.

A large inter-centre variability was noted in terms of frequency and type of AIT agents (Additional file 1: Figure S2 and S3). Beta-lactams were the agents predominantly used in 272 (76%) patients, including carbapenems (*n* = 81 (23%)). The other most frequently prescribed agents were aminoglycosides (*n* = 120 (33%)) and anti-Gram-positive agents (*n* = 51 (14%)) (Fig. 2a). The 81 patients receiving carbapenems were not different from the 278 patients receiving other AIT in terms of severity, but more frequently received combination therapy comprising aminoglycosides, anti-Gram-positive and antifungal therapies (Table 3). Interestingly, only 14/81 (17%) of these carbapenem prescriptions were documented indications.

**Table 3:** Clinical features for the patients receiving carbapenems or other antibiotics on day 0

	Missing data	Carbapenems *n* = 81	Other AIT *n* = 278	*p* value
Severity criteria at day 0				
SOFA score, median [IQR]	4/9	5 [3–7]	5 [3–7]	NS
Respiratory failure*, *n* (%)	0	32 (40)	103 (37)	NS
Cardiovascular failure*, *n* (%)	0	39 (48)	123 (44)	NS
Renal failure*, *n* (%)	0	14 (17)	53 (19)	NS
Septic shock, *n* (%)	0	29 (36)	81 (29)	NS
Acute respiratory distress syndrome, *n* (%)	0	11 (14)	26 (9)	NS
Main reasons for anti-infective therapy at day 0				
Empirical therapy, *n* (%)	0	67 (83)	232 (83)	NS
Intra-abdominal infection, *n* (%)	1/5	55 (69)	155 (57)	NS
Pneumonia, *n* (%)	2/6	7 (9)	39 (14)	NS
Bacteraemia, *n* (%)	2/3	6 (8)	39 (14)	NS
Catheter-related infection, *n* (%)	2/3	2 (3)	7 (3)	NS
Urinary tract infection, *n* (%)	2/4	–	9 (3)	NS
Skin and soft tissue infection, *n* (%)	2/3	–	2 (1)	NS
Most frequently prescribed anti-infective agents at day 0				
Beta-lactams, *n* (%)	0	81 (100)	191 (69)	< 0.001
Aminoglycosides, *n* (%)	0	41 (51)	79 (28)	< 0.001
Anti-Gram-positive agents, *n* (%)	0	20 (30)	30 (11)	< 0.01
Antifungal agents, *n* (%)	0	20 (25)	33 (12)	< 0.01
Azoles, *n* (%)	0	18 (22)	28 (10)	< 0.01
Echinocandins, *n* (%)	0	1 (1)	4 (1)	NS
Duration of AIT, days, median [IQR]	3/8	7 [3–15]	4 [1–10]	< 0.01
ICU length of stay, days, median [IQR]	0	14 [4–27]	8 [3–19]	< 0.05
ICU readmission, *n* (%)	0/1	5 (6)	22 (8)	NS
Hospital mortality, *n* (%)	0	22 (27)	71 (26)	NS
Time to death, days, median [IQR]	0	31 [7–43]	3 [1–19]	< 0.01

*NS* non-significant

*According to the definition of the SOFA score

Baseline AF therapy was reported in 53 patients (Fig. 2a, Additional file 1: Figure S2) with a predominance of azoles (*n* = 46 (13% of all AIT)) and 5 prescriptions of echinocandins. These patients did not differ from those receiving AB therapy in terms of underlying disease, initial severity or organ failure. Initial AF therapy was always administered in combination with AB therapy, mainly empirical (*n* = 36) and frequently comprising carbapenems (20/53 (38%) versus 61/306 (20%) for those receiving AB therapy, *p* < 0.05). The predominant indications for AF were intra-abdominal infections (*n* = 37 (70%)) and bacteraemia (*n* = 12 (23%)).

### Anti-infective therapy between day > 0 and day 30

Among the 359 patients receiving AIT on day 0, 19 patients did not receive any subsequent course of AIT, while the remaining 340 cases required additional AIT regimens (Figs. 1 and 2b). Overall, between day > 0 and day 30, AIT was administered to 566/860 (66%) patients, including 226 patients who were AIT-free on day 0 (Figs. 1 and 2c). No difference was observed between these two subgroups of patients in terms of underlying diseases and cause of AP. Compared to the 340 patients who received AIT from day 0, the 226 cases with delayed AIT had a less severe clinical presentation on admission. In addition, these patients subsequently experienced a more complicated course with prolonged ICU stay and prolonged AIT (Table 4 and Fig. 1).

**Table 4:** Clinical features of patients receiving AIT on admission or delayed AIT

	Missing data	Patients receiving AIT from day 0 *n* = 340	Patients receiving AIT between day > 0 and day 30 *n* = 226	*p* value
Male, *n* (%)	0/4	227 (68)	147 (65)	NS
Age, years, median [IQR]	1/1	59 [49–73]	58 [47–70]	NS
Clinical presentation on day 0				
Delay pain/ICU admission, days, median [IQR]	0	4 [1–11]	2 [1–5]	< 0.001
Oliguria/anuria, *n* (%)	0	151 (44)	99 (44)	NS
BISAP score, median [IQR]	4/14	2 [1–2]	1 [0–2]	< 0.001
Balthazar score grade E, *n* (%)	0	196 (58)	155 (69)	< 0.05
Severity criteria on day 0				
SOFA score, median [IQR]	1/11	5 [3–7]	4 [3–6]	< 0.05
Respiratory failure*, *n* (%)	0	129 (38)	68 (30)	NS
Cardiovascular failure*, *n* (%)	0	157 (46)	40 (18)	< 0.001
Renal failure*, *n* (%)	0	65 (19)	51 (23)	NS
Septic shock, *n* (%)	0	106 (31)	4 (2)	< 0.001
Acute respiratory distress syndrome, *n* (%)	0	36 (11)	16 (7)	NS
Therapeutic management on day 0				
Vasoactive support, *n* (%)	0/1	160 (47)	42 (19)	< 0.001
Mechanical ventilation, *n* (%)	0/4	126 (38)	57 (25)	< 0.01
Fluid loading, *n* (%)	1/9	270 (82)	169 (75)	NS
Renal replacement therapy, *n* (%)	1/7	40 (12)	20 (9)	NS
Main treatments between day > 0 and day 30				
Need for red blood cells transfusion, *n* (%)	0	113 (33)	78 (35)	NS
Duration of mechanical ventilation, days, median [IQR]	2/8	3 [0–11]	9 [0–21]	< 0.001
Vasoactive support, *n* (%)	0/1	205 (60)	126 (56)	NS
Renal replacement therapy, *n* (%)	1/0	91 (27)	85 (38)	< 0.01
Duration of RRT, days, median [IQR]	5/6	5 [2–15]	10 [3–16]	NS
Duration of AIT, days, median [IQR]	13/11	5 [2–12]	7 [3–14]	< 0.01
Main complications between day > 0 and day 30				
Acute respiratory distress syndrome, *n* (%)	0/2	100 (30)	101 (45)	< 0.001
Septic shock, *n* (%)	16/30	123 (40)	85 (40)	NS
Pancreatic necrosis, *n* (%)	2/16	211 (65)	174 (78)	< 0.01
Infected necrosis, *n* (%)	6/22	113 (36)	83 (38)	NS
Gastro-intestinal perforation, *n* (%)	9/21	28 (9)	22 (10)	NS
Vascular thrombosis, *n* (%)	5/20	50 (16)	33 (15)	NS
Acute mesenteric ischaemia, *n* (%)	7/20	35 (11)	27 (12)	NS
Intra-abdominal collection, *n* (%)	5/16	147 (45)	89 (40)	NS
Abdominal compartment syndrome, *n* (%)	4/21	30 (9)	35 (16)	< 0.05
Haemorrhage, *n* (%)	6/20	46 (14)	33 (15)	NS
Peritonitis, *n* (%)	8/22	58 (18)	25 (11)	< 0.05
Cholangitis, *n* (%)	7/18	41 (13)	16 (7)	< 0.05
Digestive fistula, *n* (%)	8/20	21 (7)	9 (4)	NS
Clinical management between day > 0 and day 30				
Endoscopic necrosectomy, *n* (%)	2/4	83 (25)	64 (29)	NS
Surgical necrosectomy, *n* (%)	2/4	131 (39)	86 (38)	NS
Radiological drainage, *n* (%)	2/4	72 (21)	47 (21)	NS
Duration of ICU stay, days, median [IQR]	0	8 [3–22]	17 [7–34]	< 0.001
ICU readmission, *n* (%)	0/1	27 (8)	23 (10)	NS
Hospital mortality rate, *n* (%)	0	91 (27)	50 (22)	NS
Time to death, days, median [IQR]	0	8 [1–31]	17 [5–38]	< 0.05

*NS* non-significant, *ERCP* endoscopic retrograde cholangiopancreatography

*According to the definition of the SOFA score

Most patients received AB, with a marked variability between centres (median 76% (range 54–91) of patients). The indications for AIT varied among centres, but the predominant indications remained intra-abdominal and pulmonary infections (Fig. 2b, c and Additional file 1: Figure S1, S2, and S3). Mixed sources of infections were reported in 98/340 (29%) patients treated on day 0 and receiving additional AIT before day 30 and 84/226 (37%) patients receiving delayed therapy after ICU admission (Fig. 2b, c).

Carbapenems and anti-Gram-positive agents were frequently prescribed between day > 0 and day 30, representing 202/566 (36%) and 161/566 (28%) of all AIT prescriptions, respectively (Fig. 2b, c). Patients receiving carbapenems between day 0 and day 30 had more severe disease than those treated by other AITs. These patients received more combination AIT and had a higher level of therapeutic management than those receiving other AIT, including endoscopic/radiological and surgical drainage (Table 5).

**Table 5:** Clinical features analysed for the patients receiving carbapenem or other AIT between day > 0 and day 30

	Missing data	Carbapenems *n* = 202	Other AIT *n* = 364	*p* value
Severity criteria on admission				
SOFA score, median [IQR]	4/8	5 [3–7]	5 [3–6]	NS
Respiratory failure*, *n* (%)	0	74 (37)	123 (34)	NS
Cardiovascular failure*, *n* (%)	0	79 (39)	118 (32)	NS
Renal failure*, *n* (%)	0	39 (19)	77 (21)	NS
Septic shock, *n* (%)	0	45 (22)	65 (18)	NS
Acute respiratory distress syndrome, *n* (%)	0	27 (13)	25 (7)	< 0.05
Main treatments between day > 0 and day 30				
Need for red blood cell transfusion, *n* (%)	0	87 (43)	104 (29)	< 0.001
Number of days of mechanical ventilation	5/5	12 [2–26]	2 [0–10]	< 0.001
Vasoactive support, *n* (%)	0/1	147 (73)	184 (51)	< 0.001
Renal replacement therapy, *n* (%)	0/1	82 (41)	94 (26)	< 0.001
Duration of RRT, days, median [IQR]	6/5	10 [3–22]	5 [2–13]	< 0.01
Main complications between day > 0 and day 30				
Acute respiratory distress syndrome, *n* (%)	1/1	101 (50)	100 (28)	< 0.001
Septic shock, *n* (%)	15/31	102 (55)	106 (32)	< 0.001
Pancreatic necrosis, *n* (%)	4/14	156 (79)	229 (65)	< 0.001
Infected necrosis, *n* (%)	8/20	96 (49)	100 (29)	< 0.001
Gastro-intestinal perforation, *n* (%)	12/18	23 (12)	27 (8)	NS
Vascular thrombosis, *n* (%)	9/16	36 (19)	47 (14)	NS
Acute mesenteric ischaemia, *n* (%)	10/17	26 (14)	36 (10)	NS
Intra-abdominal collection, *n* (%)	6/15	105 (54)	131 (38)	< 0.001
Abdominal compartment syndrome, *n* (%)	9/17	33 (17)	32 (9)	< 0.01
Haemorrhage, *n* (%)	8/18	44 (23)	35 (10)	< 0.001
Peritonitis, *n* (%)	11/19	36 (19)	47 (14)	NS
Cholangitis, *n* (%)	8/17	18 (9)	39 (11)	NS
Digestive fistula, *n* (%)	10/18	11 (6)	19 (5)	NS
Clinical management between day > 0 and day 30				
Endoscopic necrosectomy, *n* (%)	1/5	76 (38)	71 (20)	< 0.001
Surgical necrosectomy, *n* (%)	1/5	94 (47)	123 (34)	< 0.01
Radiological drainage, *n* (%)	1/5	63 (31)	56 (16)	< 0.001
Main reasons for anti-infective therapy between day > 0 and day 30				
Intra-abdominal infection, *n* (%)	11/13	153 (80)	194 (55)	< 0.001
Pneumonia, *n* (%)	11/14	79 (41)	89 (25)	< 0.001
Bacteraemia, *n* (%)	10/15	60 (31)	76 (22)	< 0.05
Anti-infective therapy administered between day > 0 and day 30				
Duration of AIT, days, median [IQR]	8/16	10 [3–17]	5 [2–10]	< 0.001
Aminoglycosides, *n* (%)	4/19	129 (65)	118 (34)	< 0.001
Anti-Gram-positive agents, *n* (%)	10/23	101 (53)	60 (18)	< 0.001
Antifungal agents, *n* (%)	0/2	54 (27)	56 (15)	< 0.01
Azoles, *n* (%)	6/2	32 (16)	35 (10)	< 0.05
Echinocandins, *n* (%)	6/2	24 (12)	20 (6)	< 0.01
Duration of ICU stay, days, median [IQR]	0	22 [9–39]	8 [3–19]	< 0.001
ICU readmission *n* (%)	1/0	21 (10)	29 (8)	NS
Hospital mortality rate, *n* (%)	0	64 (32)	77 (21)	< 0.01
Time to death, days, median [IQR]	0	29 [11–45]	4 [1–18]	< 0.001

*NS* non-significant, *NA* not applicable, *ERCP* endoscopic retrograde cholangiopancreatography

*According to the definition of the SOFA score

Antifungal therapy was reported in 110/566 (19%) patients with a predominance of azoles (*n* = 67 (61%)) for intra-abdominal indications (*n* = 76 (70%)) with a large between-centre variability (median of 11% of cases (range 0–31%) (Additional file 1: Figure S2).

A marked between-centre variability of therapeutic interventions was also reported between day > 0 and day 30, illustrated by large variations in the proportions of patients who underwent endoscopic necrosectomy, surgical necrosectomy and percutaneous drainages (Additional file 2: Figure S4).

### Outcome

Patients receiving AIT from ICU admission had more infectious complications and therapeutic interventions (both medical and surgical) during the first 30 days of care than AIT-free patients on day 0 (Table 2). The 226 cases with delayed AIT had more complications, increased rates of medical management (prolonged duration of mechanical ventilation and AIT, increased frequency of renal replacement therapy) and increased duration of ICU stay, but similar proportions of surgical management compared to the 340 patients who received AIT from day 0 (Table 4). In addition, the patients receiving carbapenems between day > 0 and day 30 had more infectious complications and more therapeutic interventions (both medical and surgical) than the 364 cases receiving other AITs (Table 5).

Overall, 197/860 (23%) patients died after a median interval of 4 [1–27] days. The hospital mortality rate was not significantly different between patients receiving AIT and AIT-free patients between day 0 and day 30 (Table 1). Fifty-four (20%) of these AIT-free patients died after a median [IQR] of 1.5 [1, 2] days. Most of these patients had underlying cardiovascular diseases (*n* = 42 (78%)) and were admitted for severe AP (Balthazar score E *n* = 42 (78%)). From ICU admission, they received mechanical ventilation (*n* = 27 (50%)), vasoactive support (*n* = 26 (48%)) and renal replacement therapy (*n* = 12 (22%)). Major events in the clinical course of these patients included pancreatic necrosis (*n* = 45 (87%)), mesenteric ischaemia (*n* = 22 (42%)), abdominal compartment syndrome (*n* = 9 (17%)) and/or vascular thrombosis (*n* = 8 (15%)).

Mortality rates were not different between patients treated on ICU admission and those receiving delayed AIT (Table 4). Interestingly, the outcome of patients receiving carbapenems on day 0 was not different from that of those receiving other AITs (Table 3), while patients receiving carbapenems between day > 0 and day 30 had a poorer outcome with a higher mortality rate (Table 5). Among the 205 patients with infected pancreatic necrosis, 56 (27%) died after a median [IQR] of 31 [10–44] days compared to 4 [1–19] days for the 141 patients who died without pancreatic necrosis (*p* < 0.001). A marked between-centre variability of mortality rates (range 0–41%) was reported, illustrated by 3 centres with mortality rates higher than 35% and 3 centres with mortality rates lower than 10% (Additional file 1: Figure S5). Comparison of the clinical presentation, management and outcome of the patients admitted to these units is presented in Additional file 3: Table S2.

**Fig. 3:**
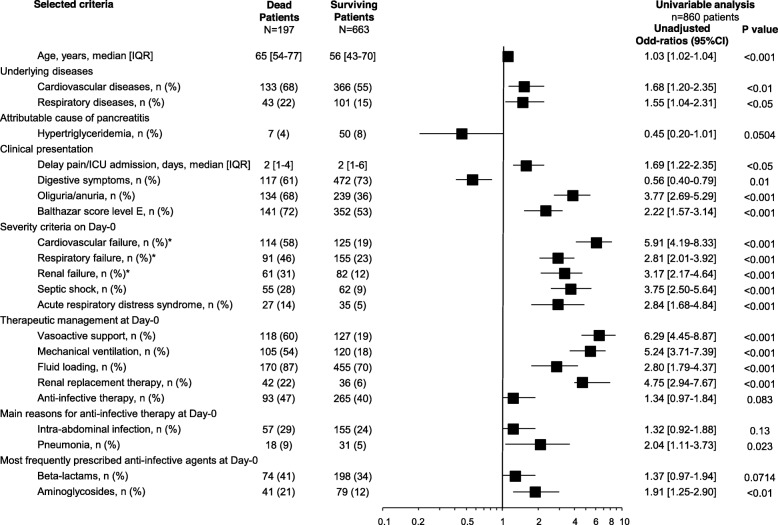
Risk factors of death observed on ICU admission (day 0) in univariate analysis in 860 complete cases

**Fig. 4:**
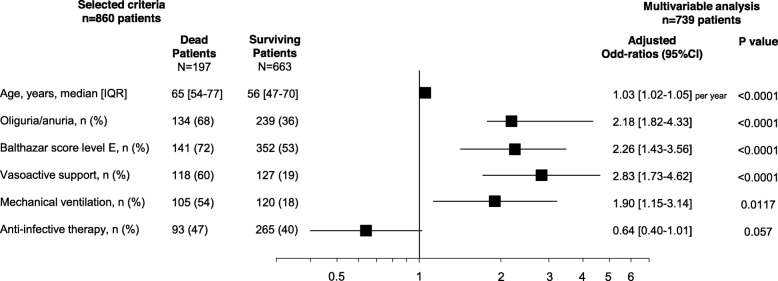
Risk factors of death observed on ICU admission (day 0) in multivariate analysis in 739 complete cases. Centre was included in the multivariate analysis as a categorical variable. *C* index, 0.83 (0.797–0.863); Hosmer-Lemeshow Test, *χ*^2^ 19.576, *p* value = 0.012

In univariate analysis, a higher mortality rate was observed among patients with septic shock or pneumonia on day 0, while AIT on day 0 did not influence the mortality rate (Fig. 3). In multivariate analysis, risk factors collected on admission and related to death were age, Balthazar score E, oliguria-anuria, vasoactive support and mechanical ventilation on admission, but not AIT (Fig. 4).

## Discussion

To the best of our knowledge, this is currently the largest study assessing the use of AIT in ICU patients admitted for the management of AP. Overall, 42% of these patients received AIT on admission. Between day > 0 and day 30, 95% of these patients received an additional course of AIT, and 45% of patients who were AIT-free on admission subsequently received AIT. Our data suggest that patients who received early AIT at day 0 presented more severe disease than patients without AIT. In addition, patients receiving delayed AIT appeared to present a higher morbidity rate, despite the absence of significantly increased mortality. Major between-centre variability was observed in terms of both the incidence of infections and therapeutic management. Underlying diseases and baseline severity appeared to be the key drivers of hospital mortality rather than infection and AIT.

In our study population, heterogeneity of practice is illustrated by major between-centre variability in terms of the incidence of infections, management of AIT and therapeutic interventions and prognosis, highlighting the need to more clearly define the indications for and modalities of AIT in AP patients and to analyse the results of treatment very carefully.

Recent guidelines do not recommend AB prophylaxis for prevention of infection of pancreatic necrosis [2]. AB prophylaxis has been extensively described in multicentre studies. In the EPIC-II study reporting the prevalence of infection in ICU patients, de Waele et al. observed prophylaxis in 24% of AP patients receiving AB [9]. In an Indian multicentre study comprising 24% of ICU cases, 67% of patients received AB, including AB prophylaxis in two thirds of cases [16]. Interestingly, prophylaxis was not reported in our cohort, which could be at least partially related to the 2001 French consensus recommendations discouraging its use in AP [17]. More recently, several studies [18, 19] and meta-analyses [20, 21] did not demonstrate any benefit for prophylaxis, which might also have influenced the prescribers’ decisions. Consequently, our data provide an interesting opportunity to assess the impact of AIT with curative intent on the outcome of ICU cases of AP.

The proportion of patients receiving AIT at the time of ICU admission was similar to that reported in the EPIC-II trial, in which 45% of patients received antibiotic therapy during the first week after admission to the ICU [9]. In a recent British national review analysing AB use for AP at the hospital level, AB therapy was administered to 62% of patients, while second and third courses were reported in 41 and 24% of cases, respectively [22]. The proportion of cases treated for intra-abdominal infection on ICU admission was much higher in our cohort than in the EPIC cohort (less than 30% in the first week of ICU stay) and the frequency of pneumonia on ICU admission in our cohort was similar to the rate reported by de Waele et al. (44/116 (28%) cases) [9]. In another large cohort of 173 infected patients with AP, Besselink et al. reported 98 (57%) cases of infected necrosis and 84 (49%) cases of pneumonia, but these rates were reported for the entire stay [23]. In another retrospective study focusing on extrapancreatic complications in ICU patients with AP, infectious complications were observed in 56/103 (54%) patients with a predominance of respiratory and urinary tract infections (43% and 21.5% of all infectious complications, respectively) [6].

The large use of carbapenems in our cohort is not surprising. The broad spectrum of these drugs and their diffusion in pancreatic tissue could at least partially explain the choice of these agents [24]. Additional explanations for these findings could be related to case-mix, local epidemiology, and surgical and endoscopic practices. In a British survey, carbapenems were the agents most commonly used in patients receiving a second course of antibiotics [22]. In line with the guidelines of the Surviving Sepsis Campaign, the use of carbapenems could be considered to be a marker of severity [15]. The selection pressure related to the extensive use of carbapenems has been previously reported to be a risk factor for the emergence of multidrug resistant organisms in many conditions [25, 26]. In the current context of dissemination of multidrug resistant Gram-negative bacilli, cautious use of carbapenems could be proposed in many cases. However, local epidemiology remains a key issue in this setting.

A limited number of AF treatments were administered both on admission and during the ICU stay. De Waele et al. reported low proportions of fungal infections [9], while other authors have suggested a growing role of fungal infections in AP. However, the patient profiles reported in the literature are quite different, marked by prolonged antibiotic therapy and ICU stay [27, 28], conditions rarely observed in our patients. Most of our cases received azoles in the context of both documented and empirical AF therapy, which could be explained by the fact that these data were collected several years ago and the proportion of echinocandins may have increased over recent years. However, the diffusion of echinocandins into necrotic pancreatic tissues needs to be formally demonstrated.

The mortality rate in our cohort was similar to that previously reported [6, 8, 9, 23]. Patients who received delayed AIT had poorer outcomes in terms of morbidity, nosocomial infections and length of ICU stay, despite a less severe clinical status on admission. Interestingly, AIT-free patients had similar mortality rates to those receiving AIT related to non-infectious complications. The role of infection and AIT on prognosis was not demonstrated in multivariate analysis, despite forcing these criteria into the analysis, suggesting that initial severity plays a major role.

Our study has several limitations. The retrospective nature of the study is obviously an important issue. However, prospective registries including such a large number of cases would appear to be difficult to achieve, and to the best of our knowledge, no such studies have yet been published. The lack of information on AIT before ICU admission is another important limitation to understand the prescribers’ treatment decisions. The indications for AIT were left to the discretion of the attending physicians, and between-centre variability is obviously a key point. The prescriber’s choices are based on microbiologically proven or suspected sites of infection motivated by the recommendations of microbiology laboratories. In a previous paper, we have reported a similar decision-making process for initiation and management of AIT in French ICUs [29]. The high between-centre variability in terms of mortality rates is another illustration of the specific case-mix admitted to ICUs. This variability led us to adjust our multivariate analysis to take this factor into account. The adequacy of AB therapy and the pharmacokinetic issues in these severely ill patients also need to be evaluated. The complete microbiological details were not available in our cohort, and the role played by various microorganisms, such as *Enterobacteriaceae*, enterococci or yeasts, needs to be analysed in more detail. The 30-day timeframe of our analysis provides an incomplete overview of the use of AIT in these cases. This time interval may be too short to demonstrate the emergence of certain specific effects, such as fungal nosocomial infections reported by some authors after prolonged AIT or prolonged ICU stay [27]. Finally, the evaluation at hospital discharge is also a limitation, as the short hospital stay in several cases could limit the validity of our findings, as some late-onset complications may have been missed.

## Conclusion

This multicentre, retrospective analysis illustrates the challenges faced by intensivists in the management of patients admitted for AP. High proportions of these patients receive AIT, both on admission and during their ICU stay, mainly for intra-abdominal and pulmonary infections. Mixed sources of infection are additional indications of AIT during the ICU stay. A large heterogeneity is observed between centres in terms of incidence of infections, AIT prescribing practices, therapeutic management and outcome. Overall, AIT reflects the initial severity and complications of AP, but is not a risk factor for death.

## Supplementary information

**Additional file 1: Figure S1.** Proportions (expressed per centre) of patients treated for septic shock (panel A) or abdominal sepsis (B) and/or pneumonia (C) on Day-0 and between Day>0 and Day 30. (data not available for centre G). **Figure S2.** Proportions (expressed per centre) of patients receiving antibiotic agents (panel A), and antifungal agents (B) on Day-0 and between Day>0 and Day-30. **Figure S3.** Proportions (expressed per centre) of patients receiving carbapenems (panel A), aminoglycosides (B), and anti-Gram-positive agents (C) on Day-0 and between Day>0 and Day-30. **Figure S5.** Mortality rates (expressed per centre).

**Additional file 2: Figure S4.** Proportions (expressed per centre) of patients who underwent endoscopic (panel A), surgical (B), percutaneous (C) or no therapeutic intervention (D) between Day>0 and Day30.

**Additional file 3: Table S1.** Clinical features for the patients receiving AIT on Day-0 according to empirical or documented prescription. **Table S2.** Comparison of the clinical features of the patients admitted in the three ICUs with the lowest mortality rates (<10%) and the three ICUs with the highest mortality rates (>35%)

## Data Availability

The datasets generated during and/or analysed during the current study are available from the corresponding author on reasonable request.
