# Supplementary material for: Epidemiology and prognosis of anti-infective therapy in the ICU setting during acute pancreatitis: a cohort study
Source: Crit Care. 2019 Dec 5;23:393. doi: 10.1186/s13054-019-2681-5 (PMC6896276; doi:10.1186/s13054-019-2681-5)
Supplement: Supplementary file 1 — Additional file 1: Figure S1. Proportions (expressed per centre) of patients treated for septic shock (panel A) or abdominal sepsis (B) and/or pneumonia (C) on Day-0 and between Day>0 and Day 30. (data not available for centre G). Figure S2. Proportions (expressed per centre) of patients receiving antibiotic agents (panel A), and antifungal agents (B) on Day-0 and between Day>0 and Day-30. Figure S3. Proportions (expressed per centre) of patients receiving carbapenems (panel A), aminoglycosides (B), and anti-Gram-positive agents (C) on Day-0 and between Day>0 and Day-30. Figure S5. Mortality rates (expressed per centre). [file 13054_2019_2681_MOESM1_ESM.pptx]

## Slide 1
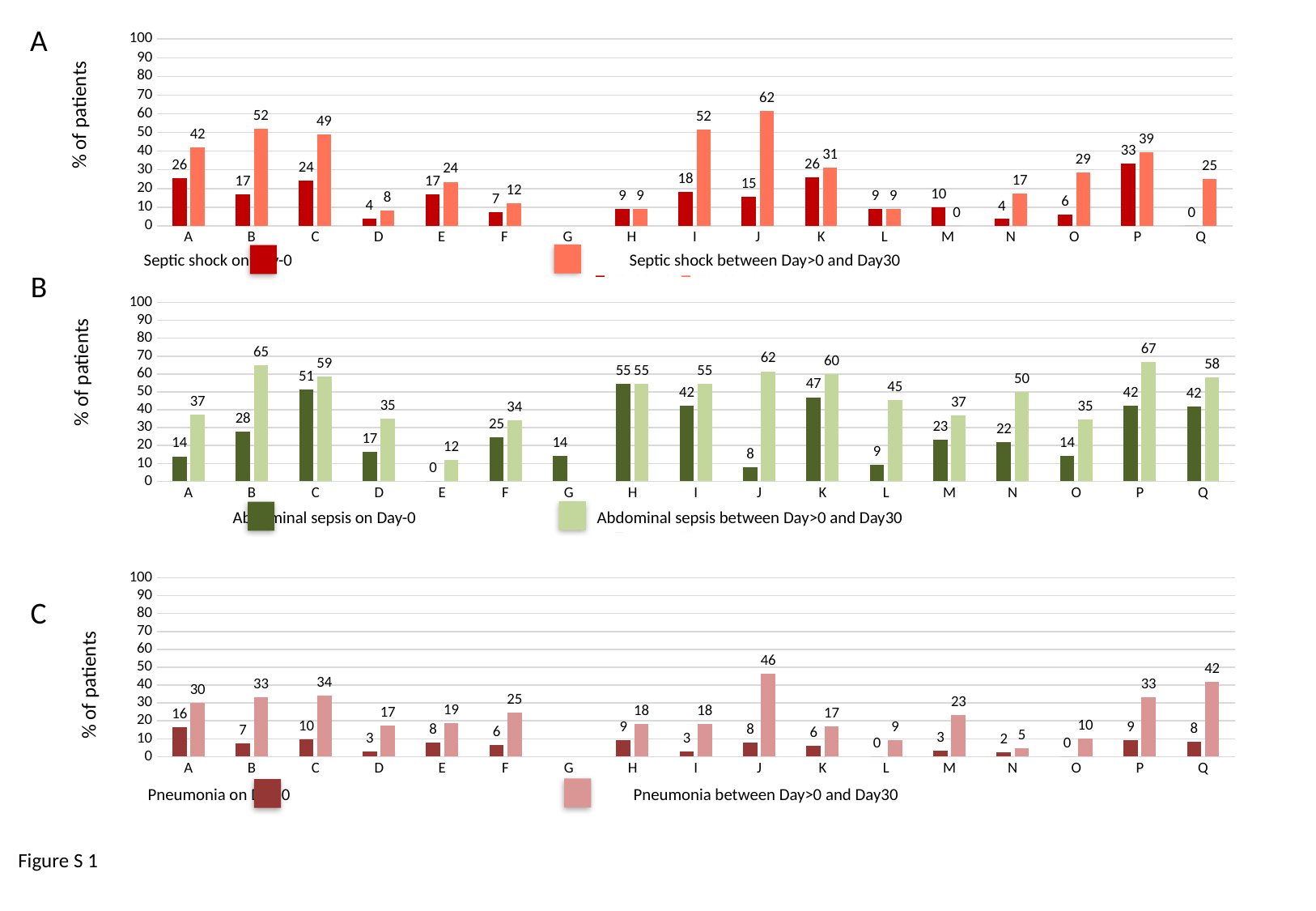

A
### Chart
| Category | %shockD0 | %ShockD30 |
|---|---|---|
| A | 25.58139534883721 | 41.86046511627907 |
| B | 16.66666666666666 | 51.85185185185183 |
| C | 24.390243902439018 | 48.78048780487805 |
| D | 3.669724770642203 | 8.256880733944959 |
| E | 16.66666666666666 | 23.52941176470588 |
| F | 7.339449541284405 | 11.926605504587162 |
| G | None | None |
| H | 9.090909090909095 | 9.090909090909095 |
| I | 18.181818181818205 | 51.51515151515152 |
| J | 15.38461538461538 | 61.53846153846154 |
| K | 26.0 | 31.0 |
| L | 9.090909090909095 | 9.090909090909095 |
| M | 10.0 | 0.0 |
| N | 3.6585365853658542 | 17.073170731707307 |
| O | 6.1224489795918355 | 28.57142857142857 |
| P | 33.33333333333333 | 39.393939393939405 |
| Q | 0.0 | 25.0 |% of patients
Septic shock on Day-0			Septic shock between Day>0 and Day30
B
### Chart
| Category | %IAID0 | %IAID30 |
|---|---|---|
| A | 13.953488372093021 | 37.20930232558141 |
| B | 27.77777777777778 | 64.81481481481481 |
| C | 51.21951219512197 | 58.53658536585365 |
| D | 16.513761467889914 | 34.86238532110093 |
| E | 0.0 | 11.76470588235294 |
| F | 24.770642201834853 | 33.944954128440365 |
| G | 14.285714285714292 | None |
| H | 54.54545454545454 | 54.54545454545454 |
| I | 42.424242424242394 | 54.54545454545454 |
| J | 7.692307692307692 | 61.53846153846154 |
| K | 47.0 | 60.0 |
| L | 9.090909090909095 | 45.454545454545425 |
| M | 23.333333333333314 | 36.666666666666636 |
| N | 21.951219512195117 | 50.0 |
| O | 14.285714285714292 | 34.69387755102039 |
| P | 42.424242424242394 | 66.66666666666664 |
| Q | 41.66666666666664 | 58.33333333333334 |% of patients
Abdominal sepsis on Day-0 		Abdominal sepsis between Day>0 and Day30
### Chart
| Category | %REspD0 | %RespD30 |
|---|---|---|
| A | 16.27906976744186 | 30.23255813953488 |
| B | 7.4074074074074066 | 33.33333333333333 |
| C | 9.75609756097561 | 34.14634146341461 |
| D | 2.7522935779816526 | 17.431192660550465 |
| E | 7.843137254901959 | 18.627450980392165 |
| F | 6.422018348623852 | 24.770642201834853 |
| G | None | None |
| H | 9.090909090909095 | 18.181818181818205 |
| I | 3.0303030303030294 | 18.181818181818205 |
| J | 7.692307692307692 | 46.15384615384611 |
| K | 6.0 | 17.0 |
| L | 0.0 | 9.090909090909095 |
| M | 3.333333333333333 | 23.333333333333314 |
| N | 2.4390243902439015 | 4.878048780487807 |
| O | 0.0 | 10.204081632653057 |
| P | 9.090909090909095 | 33.33333333333333 |
| Q | 8.333333333333332 | 41.66666666666664 |C
% of patients
Pneumonia on Day-0 	 		Pneumonia between Day>0 and Day30
Figure S 1

## Slide 2
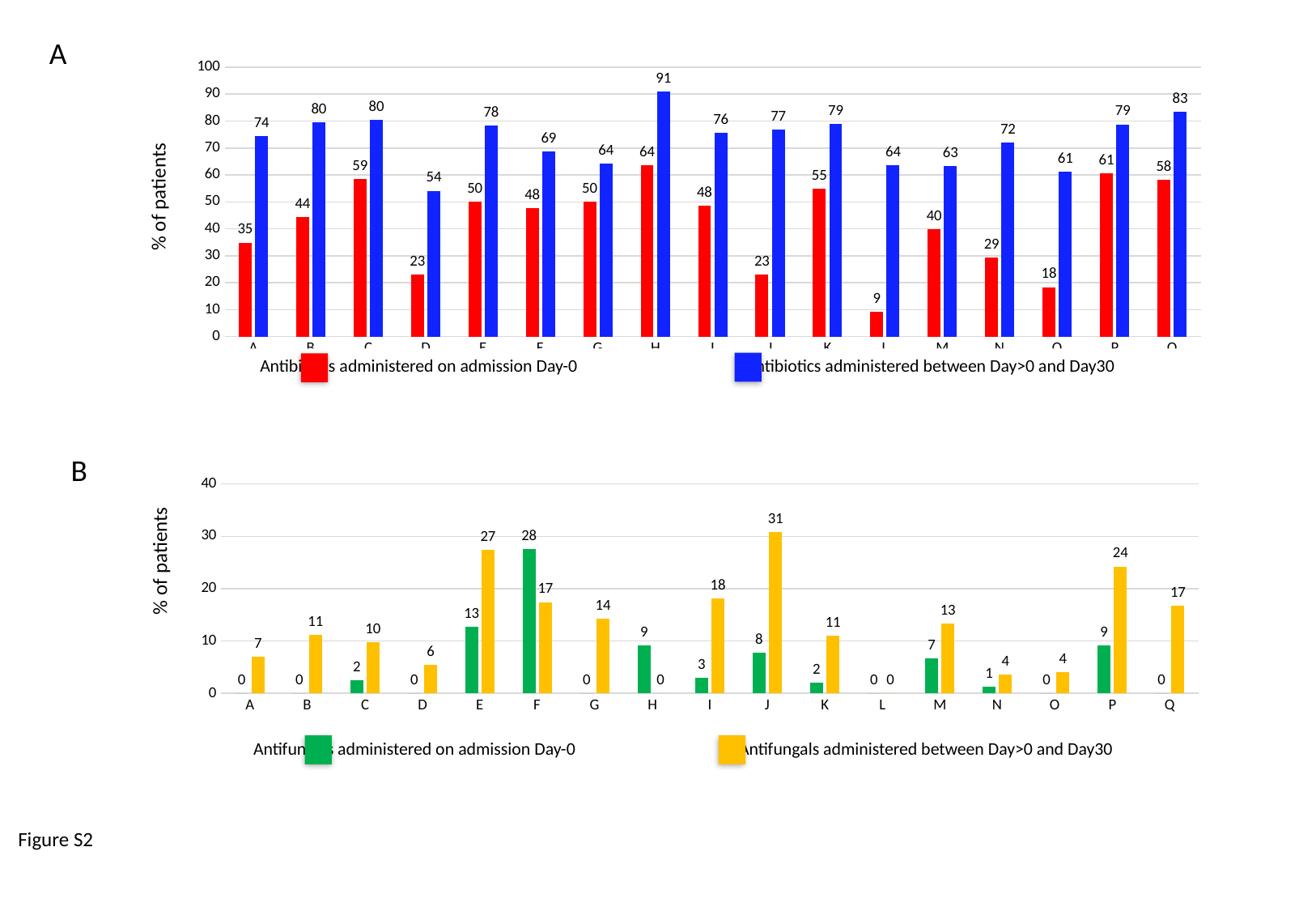

A
### Chart
| Category | %AB on admission | %AB Day30 |
|---|---|---|
| A | 34.88372093023254 | 74.41860465116281 |
| B | 44.444444444444414 | 79.62962962962963 |
| C | 58.53658536585365 | 80.4878048780488 |
| D | 22.935779816513758 | 54.128440366972484 |
| E | 50.0 | 78.43137254901961 |
| F | 47.706422018348626 | 68.8073394495413 |
| G | 50.0 | 64.2857142857143 |
| H | 63.636363636363626 | 90.9090909090909 |
| I | 48.484848484848456 | 75.75757575757575 |
| J | 23.076923076923066 | 76.9230769230769 |
| K | 55.00000000000001 | 79.0 |
| L | 9.090909090909095 | 63.636363636363626 |
| M | 40.0 | 63.33333333333333 |
| N | 29.268292682926813 | 71.95121951219512 |
| O | 18.3673469387755 | 61.22448979591835 |
| P | 60.606060606060595 | 78.78787878787878 |
| Q | 58.33333333333334 | 83.33333333333331 |% of patients
Antibiotics administered on admission Day-0 		Antibiotics administered between Day>0 and Day30
### Chart
| Category | %AF on admission | %AF Day 30 |
|---|---|---|
| A | 0.0 | 6.9767441860465125 |
| B | 0.0 | 11.111111111111109 |
| C | 2.4390243902439015 | 9.75609756097561 |
| D | 0.0 | 5.504587155963304 |
| E | 12.745098039215693 | 27.45098039215686 |
| F | 27.522935779816525 | 17.431192660550465 |
| G | 0.0 | 14.285714285714292 |
| H | 9.090909090909095 | 0.0 |
| I | 3.0303030303030294 | 18.181818181818205 |
| J | 7.692307692307692 | 30.76923076923077 |
| K | 2.0 | 11.0 |
| L | 0.0 | 0.0 |
| M | 6.666666666666667 | 13.33333333333333 |
| N | 1.219512195121951 | 3.6585365853658542 |
| O | 0.0 | 4.081632653061227 |
| P | 9.090909090909095 | 24.24242424242423 |
| Q | 0.0 | 16.66666666666666 |B
% of patients
Antifungals administered on admission Day-0 		Antifungals administered between Day>0 and Day30
Figure S2

## Slide 3
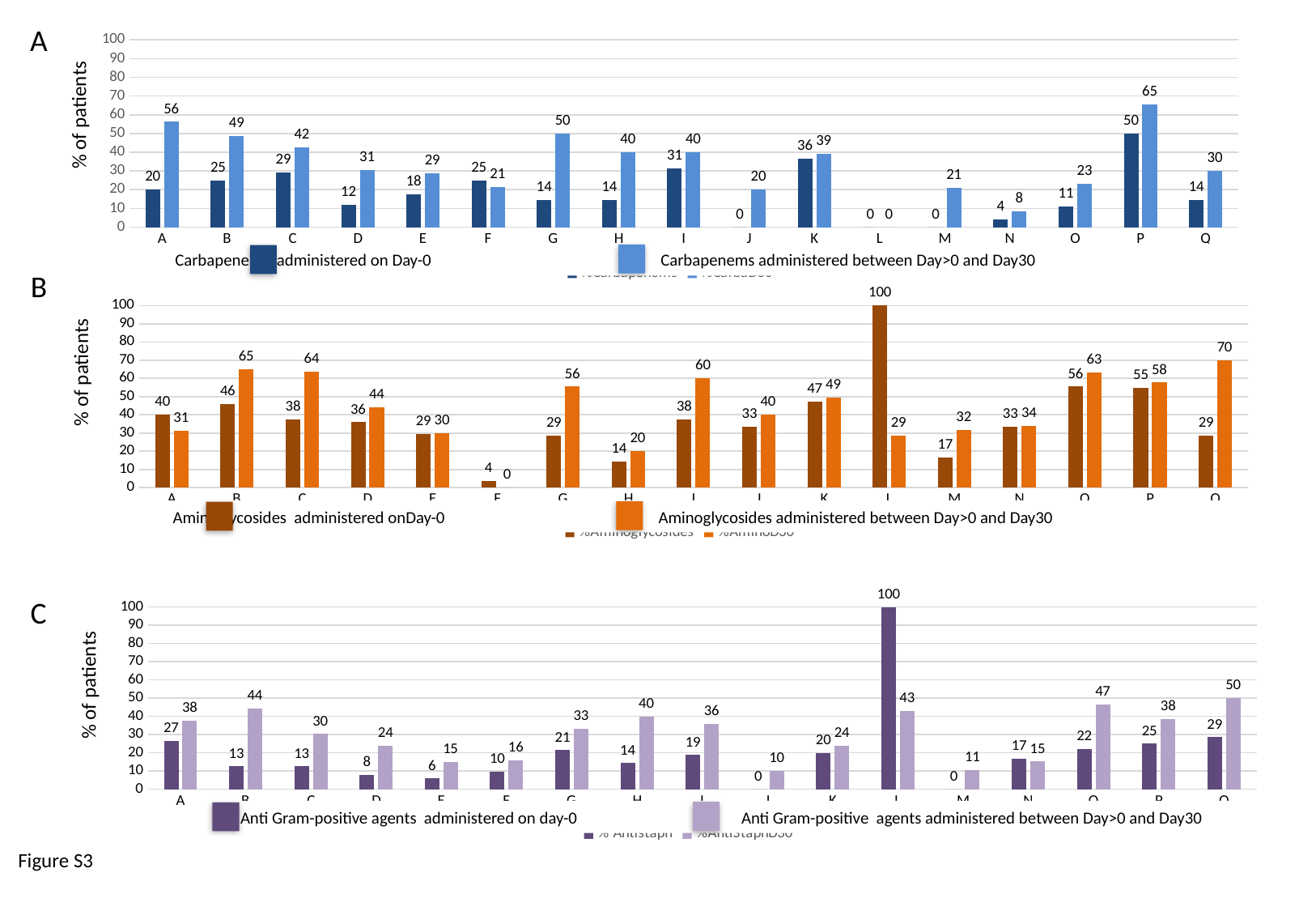

A
### Chart
| Category | %Carbapenems | %CarbaD30 |
|---|---|---|
| A | 20.0 | 56.25 |
| B | 25.0 | 48.837209302325576 |
| C | 29.16666666666667 | 42.424242424242394 |
| D | 12.0 | 30.508474576271176 |
| E | 17.647058823529424 | 28.74999999999999 |
| F | 25.0 | 21.333333333333325 |
| G | 14.285714285714292 | 50.0 |
| H | 14.285714285714292 | 40.0 |
| I | 31.25 | 40.0 |
| J | 0.0 | 20.0 |
| K | 36.36363636363634 | 39.24050632911393 |
| L | 0.0 | 0.0 |
| M | 0.0 | 21.052631578947352 |
| N | 4.166666666666666 | 8.474576271186441 |
| O | 11.111111111111109 | 23.333333333333314 |
| P | 50.0 | 65.38461538461542 |
| Q | 14.285714285714292 | 30.0 |% of patients
Carbapenems administered on Day-0 		Carbapenems administered between Day>0 and Day30
B
### Chart
| Category | %Aminoglycosides | %AminoD30 |
|---|---|---|
| A | 40.0 | 31.25 |
| B | 45.83333333333333 | 65.11627906976746 |
| C | 37.5 | 63.636363636363626 |
| D | 36.0 | 44.06779661016949 |
| E | 29.411764705882362 | 30.0 |
| F | 3.8461538461538454 | 0.0 |
| G | 28.57142857142857 | 55.55555555555556 |
| H | 14.285714285714292 | 20.0 |
| I | 37.5 | 60.0 |
| J | 33.33333333333333 | 40.0 |
| K | 47.27272727272727 | 49.36708860759492 |
| L | 100.0 | 28.57142857142857 |
| M | 16.66666666666666 | 31.578947368421048 |
| N | 33.33333333333333 | 33.898305084745765 |
| O | 55.55555555555556 | 63.33333333333333 |
| P | 55.00000000000001 | 57.69230769230769 |
| Q | 28.57142857142857 | 70.0 |% of patients
Aminoglycosides administered onDay-0		Aminoglycosides administered between Day>0 and Day30
C
### Chart
| Category | % Antistaph | %AntiStaphD30 |
|---|---|---|
| A | 26.66666666666667 | 37.5 |
| B | 12.5 | 44.18604651162789 |
| C | 12.5 | 30.303030303030297 |
| D | 8.0 | 23.728813559322024 |
| E | 5.882352941176468 | 15.0 |
| F | 9.615384615384622 | 16.0 |
| G | 21.428571428571427 | 33.33333333333333 |
| H | 14.285714285714292 | 40.0 |
| I | 18.75 | 36.0 |
| J | 0.0 | 10.0 |
| K | 20.0 | 24.05063291139239 |
| L | 100.0 | 42.85714285714284 |
| M | 0.0 | 10.526315789473678 |
| N | 16.66666666666666 | 15.25423728813559 |
| O | 22.222222222222204 | 46.666666666666636 |
| P | 25.0 | 38.46153846153845 |
| Q | 28.57142857142857 | 50.0 |% of patients
Anti Gram-positive agents administered on day-0 		 Anti Gram-positive agents administered between Day>0 and Day30
Figure S3

## Slide 4
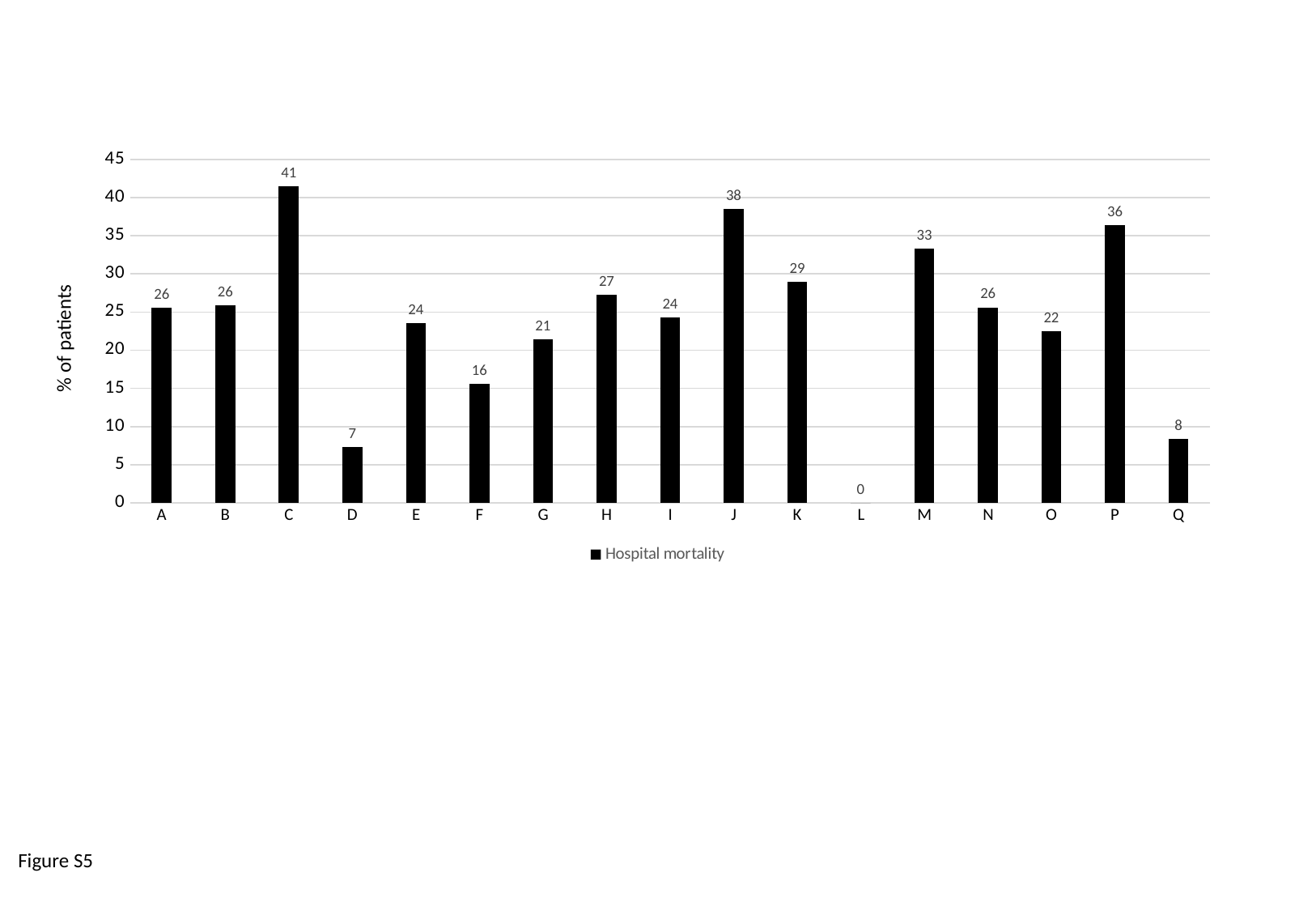

### Chart
| Category | Hospital mortality |
|---|---|
| A | 25.581395348837212 |
| B | 25.92592592592592 |
| C | 41.46341463414633 |
| D | 7.339449541284405 |
| E | 23.52941176470588 |
| F | 15.596330275229361 |
| G | 21.428571428571427 |
| H | 27.27272727272726 |
| I | 24.242424242424235 |
| J | 38.46153846153846 |
| K | 28.999999999999993 |
| L | 0.0 |
| M | 33.33333333333333 |
| N | 25.60975609756097 |
| O | 22.448979591836732 |
| P | 36.36363636363635 |
| Q | 8.333333333333332 |% of patients
Figure S5
