# Supplementary material for: Epidemiology and prognosis of anti-infective therapy in the ICU setting during acute pancreatitis: a cohort study
Source: Crit Care. 2019 Dec 5;23:393. doi: 10.1186/s13054-019-2681-5 (PMC6896276; doi:10.1186/s13054-019-2681-5)
Supplement: Supplementary file 2 — Additional file 2: Figure S4. Proportions (expressed per centre) of patients who underwent endoscopic (panel A), surgical (B), percutaneous (C) or no therapeutic intervention (D) between Day>0 and Day30. [file 13054_2019_2681_MOESM2_ESM.pptx]

## Slide 1
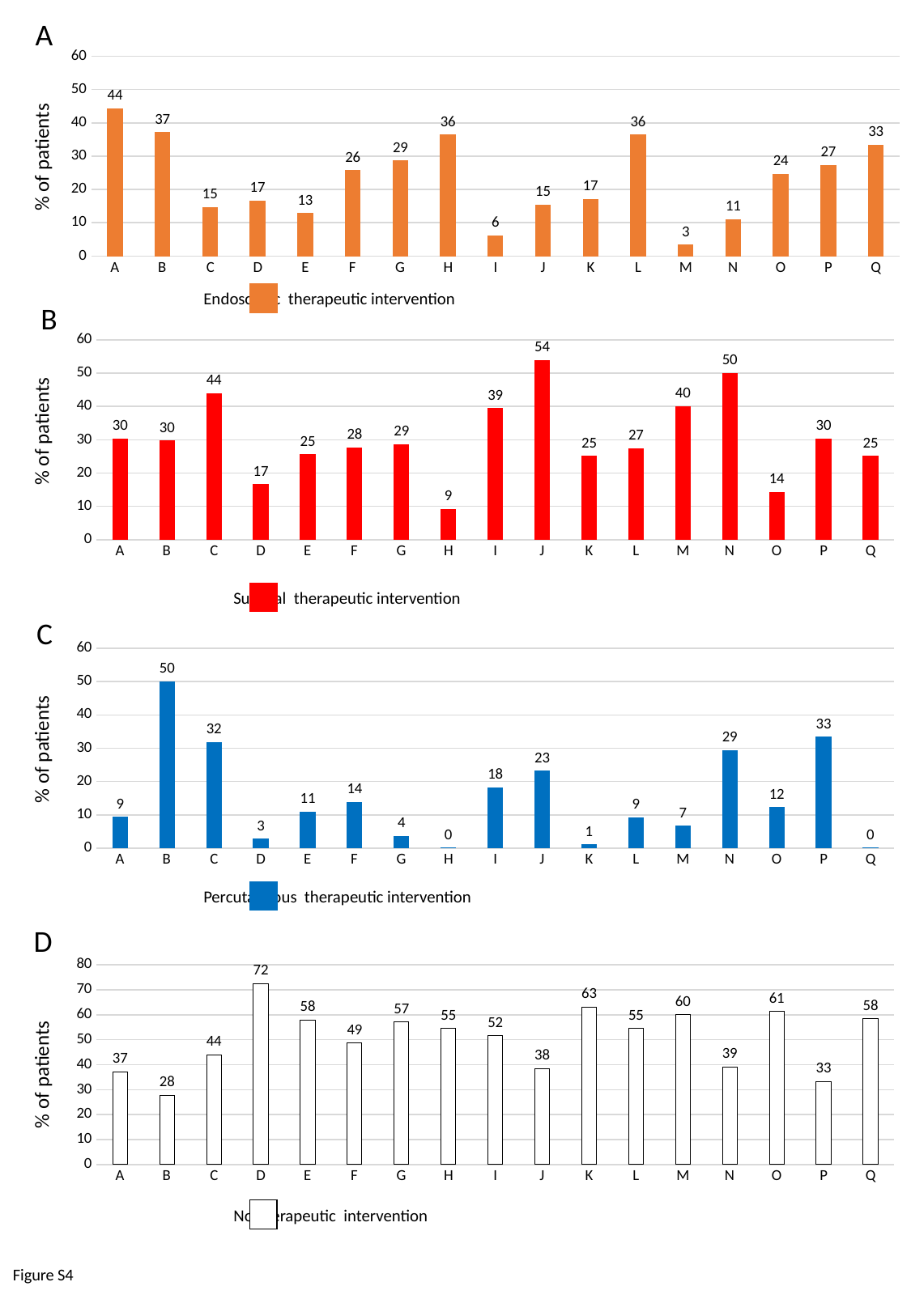

A
### Chart
| Category | % Endoscopy |
|---|---|
| A | 44.1860465116279 |
| B | 37.03703703703703 |
| C | 14.634146341463413 |
| D | 16.513761467889914 |
| E | 12.745098039215685 |
| F | 25.688073394495415 |
| G | 28.57142857142857 |
| H | 36.36363636363635 |
| I | 6.0606060606060606 |
| J | 15.384615384615385 |
| K | 17.0 |
| L | 36.36363636363635 |
| M | 3.3333333333333335 |
| N | 10.975609756097564 |
| O | 24.48979591836734 |
| P | 27.27272727272726 |
| Q | 33.33333333333333 |% of patients
Endoscopic therapeutic intervention
B
### Chart
| Category | % Surgery |
|---|---|
| A | 30.23255813953488 |
| B | 29.629629629629626 |
| C | 43.90243902439025 |
| D | 16.513761467889914 |
| E | 25.490196078431364 |
| F | 27.522935779816518 |
| G | 28.57142857142857 |
| H | 9.090909090909093 |
| I | 39.3939393939394 |
| J | 53.84615384615386 |
| K | 25.0 |
| L | 27.27272727272726 |
| M | 40.0 |
| N | 50.0 |
| O | 14.285714285714286 |
| P | 30.3030303030303 |
| Q | 25.0 |% of patients
Surgical therapeutic intervention
C
### Chart
| Category | % Radiology |
|---|---|
| A | 9.30232558139535 |
| B | 50.0 |
| C | 31.707317073170724 |
| D | 2.752293577981652 |
| E | 10.784313725490193 |
| F | 13.761467889908259 |
| G | 3.5714285714285707 |
| H | 0.0 |
| I | 18.18181818181819 |
| J | 23.076923076923073 |
| K | 1.0 |
| L | 9.090909090909093 |
| M | 6.666666666666667 |
| N | 29.26829268292682 |
| O | 12.244897959183673 |
| P | 33.33333333333333 |
| Q | 0.0 |% of patients
Percutaneous therapeutic intervention
D
### Chart
| Category | No procedure |
|---|---|
| A | 37.20930232558141 |
| B | 27.77777777777778 |
| C | 43.90243902439025 |
| D | 72.4770642201835 |
| E | 57.84313725490197 |
| F | 48.623853211009184 |
| G | 57.14285714285714 |
| H | 54.54545454545454 |
| I | 51.515151515151516 |
| J | 38.46153846153846 |
| K | 63.0 |
| L | 54.54545454545454 |
| M | 60.0 |
| N | 39.02439024390244 |
| O | 61.22448979591836 |
| P | 33.33333333333333 |
| Q | 58.333333333333336 |% of patients
No therapeutic intervention
Figure S4
