# Supplementary material for: Epidemiology and prognosis of anti-infective therapy in the ICU setting during acute pancreatitis: a cohort study
Source: Crit Care. 2019 Dec 5;23:393. doi: 10.1186/s13054-019-2681-5 (PMC6896276; doi:10.1186/s13054-019-2681-5)
Supplement: Supplementary file 3 — Additional file 3: Table S1. Clinical features for the patients receiving AIT on Day-0 according to empirical or documented prescription. Table S2. Comparison of the clinical features of the patients admitted in the three ICUs with the lowest mortality rates (<10%) and the three ICUs with the highest mortality rates (>35%) [file 13054_2019_2681_MOESM3_ESM.doc]

Table S1. Clinical features for the patients receiving AIT on Day-0 according to empirical or documented prescription

|  | Missing data | Empirical  AIT  n=299 | Documented AIT  n=60 | P value |
| --- | --- | --- | --- | --- |
| Male, n (%) | 3/1 | 195 (66) | 44 (75) | NS |
| Age, years, median [IQR] | 1/2 | 61 [49-74] | 54 [45-66] | <0.05 |
| Clinical presentation at Day-0 | | | | |
| Pain onset/ICU admission interval, days, median [IQR] | 0 | 3 [1-8] | 7 [1-16] | <0.05 |
| Oliguria/anuria, n (%) | 0 | 133 (44) | 21 (35) | NS |
| BISAP score, median [IQR] | 14/4 | 2 [1-2] | 2 [1-2] | NS |
| Balthazar score E, n (%) | 0 | 170 (57) | 31 (52) | NS |
| Severity criteria at Day-0 | | | | |
| SOFA score, median [IQR] | 12/1 | 5 [3-7] | 5 [2-7] | NS |
| Respiratory failure*, n (%) | 0 | 121 (40) | 17 (28) | NS |
| Cardiovascular failure*, n (%) | 0 | 144 (48) | 18 (30) | <0.05 |
| Renal failure*, n (%) | 0 | 44 (15) | 9 (15) | NS |
| Septic shock, n (%) | 0 | 98 (33) | 12 (20) | NS |
| Acute respiratory distress syndrome, n (%) | 0 | 33 (11) | 4 (7) | NS |
| Initial therapeutic management  Vasoactive agents, n (%) | 1/0 | 147 (49) | 18 (30) | <0.01 |
| Mechanical ventilation, n (%)  Fluid loading, n (%)  Renal replacement therapy, n (%) | 3/1  6/5  7/2 | 116 (39)  234 (80)  34 (12) | 14 (24)  44 (80)  8 (14) | <0.05  NS  NS |
| Main reasons for anti-infective therapy at Day-0 | | | | |
| Intra-abdominal infection, n (%) | 4/2 | 173 (58) | 34 (57) | NS |
| Pneumonia, n (%) | 6/2 | 41 (14) | 5 (9) | NS |
| Bacteraemia, n (%)  Catheter-related infection, n (%) | 4/1  4/1 | 28 (9)  3 (1) | 15 (25)  6 (10) | <0.01  <0.001 |
| Urinary tract infection, n (%) | 5/1 | 8 (3) | 1 (2) | NS |
| Skin and soft tissue infection, n (%) | 4/1 | 2 (1) | 0 | NS |
| Most frequently prescribed anti-infective agents at Day-0 | | | | |
| Beta-lactams, n (%) | 0 | 225 (75) | 47 (78) | NS |
| Carbapenems, n (%) | 0 | 67 (22) | 14 (23) | NS |
| Aminoglycosides, n (%) | 0 | 109 (36) | 11 (18) | <0.01 |
| Anti-Gram-positive agents, n (%) | 0 | 40 (13) | 10 (17) | NS |
| Antifungal agents, n (%)  Azoles, n (%)  Echinocandins, n (%)  Duration of AIT, days, median [IQR] | 0  0  0  9/2 | 36 (12)  31 (10)  1 (1)  5 [2-11] | 17 (28)  15 (25)  4 (7)  5 [1-10] | <0.01  <0.01  <0.01  NS |
| ICU length of stay, days, median [IQR]  ICU readmission n (%)  Hospital mortality, n (%)  Time to death, days, median [IQR] | 0  1/0  0  0 | 9 [3-21]  22 (7)  84 (28)  4 [1-32] | 7 [4-21]  5 (8)  9 (15)  8 [4-23] | NS  NS  <0.05  NS |

NS: non-significant

*According to the definition of the SOFA score

Table S2. Comparison of the clinical features of the patients admitted in the three ICUs with the lowest mortality rates (<10%) and the three ICUs with the highest mortality rates (>35%)

|  | Missing data | ICUs with low mortality rate  N=87 | ICUS with high mortality rate  N=132 | P value |
| --- | --- | --- | --- | --- |
| Clinical presentation on admission |  |  |  |  |
| BISAP on admission, median (IQR) | 0 | 1 [0-2] | 1 [1-2] | <0.01 |
| Pain onset/ICU admission interval, days median (IQR) | 0 | 2 [1-4] | 2 [1-6] | NS |
| Oliguria/anuria, n (%) | 0 | 44 (33) | 38 (44) | NS |
| Balthazar score E on admission, n (%) | 0 | 73 (55) | 69 (79) | <0.001 |
| Severity criteria on admission |  |  |  |  |
| SOFA on admission, median (IQR) | 0 | 3 [2-6] | 5 [3-6] | <0.01 |
| Respiratory failure*, n (%) | 0 | 20 (15) | 37 (43) | <0.001 |
| Cardiovascular failure*, n (%) | 0 | 13 (10) | 35 (40) | <0.001 |
| Renal failure*, n (%) | 0 | 16 (12) | 16 (18) | NS |
| Septic shock, n (%) | 0 | 5 (7) | 23 (33) | <0.001 |
| Acute respiratory distress syndrome, n (%) | 0 | 7 (5) | 16 (18) | <0.01 |
| Therapeutic management on admission |  |  |  |  |
| Vasoactive support, n (%) | 0 | 13 (10) | 36 (41) | <0.001 |
| Mechanical ventilation, n (%) | 0 | 8 (6) | 37 (43) | <0.001 |
| Renal replacement therapy, n (%) | 0 | 6 (5) | 9 (10) | NS |
| Anti-infective therapy on admission |  |  |  |  |
| Antibiotic therapy on Day-0, n (%) | 0 | 34 (25) | 46 (53) | <0.001 |
| Empirical antibiotic therapy on Day-0, n (%) | 0 | 16 (47) | 44 (96) | <0.001 |
| Beta-lactams on Day-0, n (%) | 4/1 | 33 (25) | 44 (53) | <0.001 |
| Carbapenems on Day-0, n (%) | 3/1 | 5 (4) | 16 (19) | <0.001 |
| Aminoglycosides on Day-0, n (%) | 3/1 | 13 (10) | 21 (25) | <0.01 |
| Anti-Gram-positive agents on Day-0, n (%) | 3/0 | 5 (4) | 8 (10) | NS |
| Antifungal therapy, n (%) | 0 | 0 | 5 (6) | <0.01 |
| Abdominal source of infection on Day-0, n (%) | 0 | 25 (19) | 36 (41) | <0.001 |
| Pulmonary source of infection on Day-0, n (%) | 0 | 4 (3) | 8 (9) | NS |
| Main treatments between Day>0 and Day30 |  |  |  |  |
| Need for blood transfusions, n (%) | 0 | 15 (32) | 32 (37) | <0.001 |
| Duration of mechanical ventilation, days, median (IQR) | 0 | 0 [0-6] | 6 [0-20] | <0.001 |
| Vasoactive support, n (%) | 0 | 25 (19) | 51 (59) | <0.001 |
| Renal replacement therapy, n (%) | 0 | 20 (15) | 29 (33) | <0.01 |
| Anti-infective therapy between Day>0 and Day30 | | | | |
| Duration of AIT, days, median (IQR) | 9/2 | 2 [0-7] | 6 [1-14] | <0.001 |
| Carbapenems, n (%) | 0 | 21 (16) | 33 (48) | <0.001 |
| Aminoglycosides, n (%) | 2/0 | 35 (27) | 40 (47) | <0.01 |
| Anti-Gram-positive agents, n (%) | 3/0 | 22 (17) | 21 (25) | NS |
| Antifungal agents, n (%) | 1/0 | 8 (6) | 16 (19) | <0.01 |
| Main reasons for AIT between Day>0 and Day30 | | | | |
| Intra-abdominal infection, n (%)  Pneumonia, n (%) | 1/0  1/0 | 50 (38)  25 (19) | 54 (63)  31 (36) | <0.001  <0.01 |
| Main complications between Day>0 and Day30 |  |  |  |  |
| Acute respiratory distress syndrome, n (%) | 0 | 28 (21) | 39 (45) | <0.001 |
| Septic shock, n (%) | 4/0 | 13 (10) | 41 (49) | <0.001 |
| Pancreatic necrosis, n (%) | 0 | 95 (72) | 81 (93) | <0.001 |
| Infected necrosis, n (%) | 0 | 18 (14) | 35 (40) | <0.001 |
| Gastro-intestinal perforation, n (%) | 0 | 7 (5) | 12 (14) | <0.05 |
| Acute mesenteric ischemia, n (%) | 0 | 2 (2) | 14 (16) | <0.001 |
| Intra-abdominal collection, n (%) | 1/0 | 29 (22) | 52 (60) | <0.001 |
| Abdominal compartment syndrome, n (%) | 1/0 | 21 (16) | 9 (10) | NS |
| Haemorrhage, n (%) | 1/0 | 10 (8) | 16 (19) | <0.05 |
| Peritonitis, n (%) | 1/0 | 4 (3) | 27 (31) | <0.001 |
| Clinical management between Day>0 and Day30 | | | | |
| Endoscopic necrosectomy, n (%) | 1/0 | 26 (20) | 17 (20) | NS |
| Surgical necrosectomy, n (%) | 1/0 | 24 (18) | 35 (41) | <0.001 |
| Radiological drainage, n (%) | 1/0 | 4 (3) | 27 (31) | <0.001 |
| Duration of ICU stay, days, median (IQR) | 0 | 6 [3-17] | 14 [4-28] | <0.01 |
| ICU readmission, n (%) | 0 | 6 (5) | 9 (10) | NS |
| Hospital mortality rate, n (%) | 0 | 9 (7) | 34 (39) | <0.001 |
| Time to death, days, median (IQR) | 0 | 17 [2.5-30] | 15 [2-37] | NS |

NS: non-significant

*According to the definition of the SOFA score
